# Variability of NT-proBNP and Its Relationship with Inflammatory Status in Patients with Stable Essential Hypertension: A 2-Year Follow-Up Study

**DOI:** 10.1371/journal.pone.0031189

**Published:** 2012-02-23

**Authors:** Esther Roselló-Lletí, Jose R. Calabuig, Pedro Morillas, Raquel Cortés, Luis Martínez-Dolz, Luis Almenar, Jose R. González-Juanatey, Catheline Lauwers, Antonio Salvador, Manuel Portolés, Vicente Bertomeu, Miguel Rivera

**Affiliations:** 1 Cardiocirculatory Unit, Research Center, Hospital Universitario La Fe, Valencia, Spain; 2 Internal Medicine, Hospital Universitario La Fe, Valencia, Spain; 3 Cardiology Unit, Hospital Universitario San Juan, Alicante, Spain; 4 Cardiology Unit, Hospital Universitario la Fe, Valencia, Spain; 5 Cardiology Unit, Hospital Clínico, Santiago de Compostela, Spain; 6 Cardiology Unit, Hospital San Francesc de Borja, Gandía, Spain; 7 Cell Biology and Pathology Unit, Research Center, Hospital Universitario La Fe, Valencia, Spain; I2MC INSERM UMR U1048, France

## Abstract

**Background:**

The variability of NT-proBNP levels has been studied in heart failure, yet no data exist on these changes over time in hypertensive patients. Furthermore, studies on the relationship between natriuretic peptides and inflammatory status are limited.

**Methodology/Principal Findings:**

220 clinically and functionally asymptomatic stable patients (age 59±13, 120 male) out of 252 patients with essential hypertension were followed up, and NT-proBNP was measured at baseline, 12 and 24 months. No differences in NT-proBNP were found with respect to the basal stage in the hypertrophic group, but significant changes were found in non-hypertrophic subjects. The reproducibility of NT-proBNP measurements was better in patients with hypertrophy than in the non-hypertrophic group for the three intervals (stage I-basal; stage II-stage I; stage II-basal) **with a reference change value of 34%, 35% and 41%**, respectively, in the hypertrophic group. A more elevated coefficient of correlation was obtained in the hypertrophic group than in patients without hypertrophy: basal versus stage I (r = 0.79, p<0.0001 and r = 0.59, p<0.0001) and stage I versus stage II (r = 0.86, p<0.0001 and r = 0.56, p<0.0001). Finally, levels of NT-proBNP significantly correlated with sTNF-R1 (p<0.0001) and IL-6 (p<0.01) during follow-up. A multivariate linear regression analysis showed that sTNF-R1 is an independent factor of NT-proBNP.

**Conclusions/Significance:**

This work shows that there is good stability in NT-proBNP levels in a follow-up study of asymptomatic patients with stable hypertension and left ventricular hypertrophy. As a consequence, assessment of NT-proBNP concentrations may be a useful tool for monitoring the follow-up of hypertensive patients with hypertrophy. Measured variations in peptide levels, exceeding **35%** in a 12-month follow-up and **41%** in a 24-month follow-up, may indicate an increase in cardiovascular risk, and therefore implies adjustment in the medical treatment. In addition, this study shows a link between neurohormonal and inflammatory activation in these patients.

## Introduction

Circulating levels of natriuretic peptides are elevated in states of increased cardiac wall stress. B-type natriuretic peptide (BNP) and amino-terminal propeptide of B-type natriuretic peptide (NT-proBNP) concentrations are well established markers for the diagnosis and prognosis of patients with heart failure [1]–[3]. Furthermore, the Task Force of the European Society of Cardiology for the Diagnosis and Treatment of Chronic Heart Failure recommends that a natriuretic peptide assay should be included in the first step of the algorithm for the diagnosis of heart failure together with electrocardiography and chest x ray findings, on the basis of its strong negative predictive value.

Left ventricular hypertrophy (LVH) is the main mechanism of compensation for hemodynamic overload in hypertension. It has been shown that NT-proBNP serum levels are increased in hypertensive patients with LVH [4]. This natriuretic peptide predicts cardiovascular events and is considered a marker of cardiovascular risk in the general population [5] and in patients with hypertension [6]. Furthermore, in a recent study Paget *et al.* show that this peptide is a powerful predictor of mortality in hypertensive patients without heart failure [7]. Therefore, NT-proBNP adds independent prognostic information and could be used to monitor hypertensive patients.

Knowing the variations in NT-proBNP levels before the clinical use of this peptide as a tool to monitor patients is crucial. However, there is a limited number of studies addressing natriuretic peptide variability, and these works have evaluated the biological variation of BNP and NT-proBNP concentrations in both patients with chronic heart failure and healthy people over a short (within a day, week to week and month to month) **and intermediate (1-month, 2-month, and 3-month) interval of time** [**8]**–[**11**] **.** Schou *et al.* and our group have shown in previous works the variability of NT-proBNP levels in patients with stable heart failure during a 24-month follow-up [12], [13], yet to date, there are no data on the changes in serum NT-proBNP levels over time in asymptomatic stable patients with essential hypertension. This would allow us to know the usefulness of this peptide in the clinical arena.

Several lines of evidences support a role for TNF-alpha, its soluble receptors and IL-6 as predictors of cardiovascular events [14], [15]..In addition, in a previous report, our group showed that the profile of circulating cytokines was altered in patients with essential hypertension [16]. However, studies on the relationship between inflammatory markers and NT-proBNP are limited [17], [18]. In fact, to the best of our knowledge, cytokine levels have never been correlated with NT-proBNP concentrations in hypertensive patients.

We hypothesize that NT-proBNP levels may change over time even in patients with clinically stable hypertension and this peptide could be associated with inflammatory status. Therefore, the purpose of this study was to analyze NT-proBNP variability during a 24-month follow-up, and to evaluate the relationship between NT-proBNP levels and circulating inflammatory markers (sTNF-R1 and IL-6) in a cohort of stable asymptomatic hypertensive patients.

## Methods

### Ethics statement

All patients gave written informed consent to participate in the study. The project was approved by the local Ethics Committee (Biomedical Investigation Ethics Committee of “La Fe” University Hospital of Valencia, Spain) and conducted in accordance with the guidelines of the Declaration of Helsinki.

### Patients

The study was on 252 Caucasian asymptomatic hypertensive consecutive out-patients (mean ± SD age 60±13 years, 136 male), from 11 participating hospitals. All patients underwent a routine physical examination, electrocardiogram, echo-Doppler study and laboratory analyses. Physicians using a standardized protocol measured systolic and diastolic blood pressure in the left arm of seated subjects between 08:00 and 11:00 AM, following the recommendations of The American Heart Association [19]. **Patients were included in the study between August 2007 and October 2007.** Of the 252 subjects, 220 asymptomatic (they did not refer any symptoms of cardiovascular origin, specifically any symptoms of heart failure) and stable patients (without cardiovascular events [6]) were included in the study (122 with LVH and 98 without LVH, mean ± SD age 59±13 years, 120 male). Thirty-two (13%) were excluded during follow-up (21 refused to continue, 9 could not be located, 1 patient had a stroke and 1 patient had a myocardial infarction). We decided to analyze separately patients with and without LVH, because of the differences in cardiac structure and prognosis.

Patients analyzed in this study met this inclusion criteria: a previous diagnosis of hypertension, as defined by the Seventh Report of the Joint National Committee on Prevention, Detection, Evaluation, and Treatment of High Blood Pressure [19]. Furthermore, exclusion criteria were secondary HT, left ventricular ejection fraction <50, ischemic (medical history, echo-Doppler, troponin T assay) or dilated cardiomyopathy, atrial fibrillation, more than mild valvular disease, acute and chronic liver or renal diseases, immunological diseases, HIV, alcoholism and drug addiction and any other life-threatening disease.

At least 2 months before study enrollment all patients were on stable medical therapy with angiotensin II receptor antagonist 50%, diuretics 45%, angiotensin-converting enzyme inhibitors 32%, β-blockers 21%, statins 26%, and calcium-channel blockers 19%. No statistically significant changes were observed in the different drugs administered during follow-up. None of the 220 patients finally studied presented cardiovascular events (defined as stroke, myocardial infarction or cardiovascular death) [6]. Body mass index was calculated as the weight in kilograms divided by height in meters squared, and obesity was defined as body mass index >30 kg/m^2^. Glomerular filtration rate was calculated using the modified diet in renal disease equation [20]. All patients were followed up until the end of the study at month 24, with a three-stage sample collection: basal, 12 months (stage I) and 24 months (stage II). All explorations were made in each stage.

The procedure was approved by the appropriate institutional review boards or ethics review committees of each study center, and the study was conducted in accordance with the guidelines of good clinical practice and with ethical standards for human experimentation established by the Declaration of Helsinki. Every patient signed a written informed consent for their inclusion in the study.

### NT-proBNP determination

Samples were collected under standardized conditions to minimize sources of preanalytical variation. Venous blood was taken by venipuncture with the subjects in sitting position between 08:00 and 11:00 AM, centrifuged immediately, and frozen at −80°C. After thawing, serum NT-proBNP levels were determined in a single laboratory using the commercially available Elecsys proBNP sandwich, electrochemiluminescence immunoassay on an Elecsys 2010 Analyzer (Roche Diagnostics, Mannheim, Germany). The results are expressed as pg/ml (equivalent to ng/l, SI units). The lower detection limit was 5 pg/ml, and intra-assay variation was 2.6%.

### Cytokine and cytokine receptor determination

Venus blood was taken by venipuncture into pyrogen-free vacuum tubes containing EDTA, as anticoagulant, with the subjects in sitting position between 8:00 and 11:00 AM, centrifuged immediately, frozen at −80°C and only thawed once. Plasma concentrations of sTNF-R1 and IL-6 were determined at a central laboratory by specific commercial sandwich enzyme-linked immunosorbent assays (Hbt human sTNF-R1 ELISA test kit; Hycult Biotechnology, The Netherlands; Strakine human IL-6 ELISA; Strathmann Biotec, Germany). The tests were quantified at 450 nm in a dual-wavelength microplate reader (Sunrise; TECAN, Austria) using Magellan software (version 2.5; TECAN, Austria). The sTNF-R1 and IL-6 tests have limits of detection of 25 and 0.3 pg/ml, respectively. Our intra-assay and inter-assay coefficients of variation were 6.5 and 9.1% for sTNF-R1 and 6.1 and 8.3% for IL-6, respectively.

### Echo-Doppler study

The study was performed using standard hospital echocardiographic systems equipped with 2.5–4 MHz transducers. The echocardiographic examinations were performed using the standard apical and parasternal long axis views. Doppler echocardiogram images were stored on videotape and analyses of recordings were performed in a central laboratory. M-Mode and two-dimensional images, Doppler spectrum and color Doppler were analyzed off-line. For each patient, four consecutive beats were measured and averaged for each Doppler variable.

To obtain left ventricular ejection fraction, the area-length method was used [21]. Left ventricular mass was measured following the Devereux method [22] and in our study LVH was defined as >46.7 g/m^2.7^ in women and >49.2 g/m^2.7^ in men [23]. **The following measurements were used for assessment of LV diastolic dysfunction: 1) mitral flow propagation velocity (Vp) was determined using the previously described method** [**24**] **; 2) peak flow velocity in early diastole (E-wave) and during atrial contraction (A) was measured by pulsed Doppler at valve level, calculating the E/A ratio; 3) early LV filling deceleration time (DT) was measured as the distance (time) between the projection of the peak velocity on the baseline and the point where EF slope encounters the baseline; 4) the inclination of the straight line of the ascending mitral ring in M-mode recording shows maximum ascending velocity (mm/s) of the mitral annulus during early diastole. The maximum relaxation velocity (RVm) was calculated as mean value of the maximal velocities in the septal, lateral, posterior, and anterior portion of the annulus** [**25**] **.** Intra-observer variability was consecutively evaluated in series of 40 patients. Variability was expressed as the absolute difference divided by the mean value of echocardiographic measurements, left ventricular mass variability being 8.4±6%.

### Statistical analyses

Continuous variables are presented as mean ± standard deviation and categorical variables as a number of patients or percentage. Results for each variable were tested for normality using the Kolmogorov Smirnov method. NT-proBNP concentrations exhibited a non-normal distribution and were presented as the median and interquartile range and log transformed (and proved to be normalized) before parametric correlation analysis. Temporal changes in peptide levels and clinical characteristics were analyzed using the paired Student's *t* test, and categorical variable changes were compared using the McNemar test. Correlation between NT-proBNP at baseline, stage I, and stage II was also determined using Pearson's coefficient. Cytokine concentrations exhibited a non-normal distribution and were log transformed (and proved to be normalized) before parametric correlation analysis. Furthermore, multivariate linear regression analysis was performed using log-transformed NT-proBNP as dependent variable and included age, gender, treatment, **systolic and diastolic blood pressure, total cholesterol**, log-transformed sTNF-R1 and log-transformed IL-6 as independent variables. The discrimination of the best model was based on the principle of least mean square and higher R-square.

To compare NT-proBNP levels in both the LVH and non-LVH groups over two different time intervals (stage I - basal; stage II – basal; stage II – stage I), we used the statistical method of Bland Altman [26], [27]. In this graphical method the percentage of change in the averages ((NT-proBNP stage I – NT-proBNP basal)/(average stage I + basal)) is plotted against the average of the two NT-proBNP measurements. This expression is useful to normalize and compare the data without taking into account the magnitude of the NT-proBNP measurement. Based on this approach, the limits of agreement were determined by the mean difference plus or minus the coefficient of reproducibility (CR), where CR was calculated as 1.96× SD of the percentage of changes. In this case, a high CR indicates poor reproducibility. **The coefficient of variation (CV) is calculated following CV = 100 (standard deviation/mean). The total CV (CV_t_) and the analytical CV (CV_a_) provided the basis for the individual biological CV (CV_i_) following CV_i_ = (CV_t_^2^ – CV_a_^2^)^1/2^. Reference change values (RCVs) were calculated from median CV_t_ values, according to the formula RCV = Z×2^1/2^ (CV_a_^2^/** ***n*_a_+CV_i_^2^/*n*_s_)^1/2^, where Z = 1.96; *n*_a_ is the number of replicate assays; and *n*_s_ is the number of patient samples to estimate each of the two homeaostatic set points.** A *p value*<0.05 was considered significant for all measures. All statistical analyses were performed using the SPSS statistical software package (SPSS Inc., Chicago, IL).

## Results

**Figure 1:**
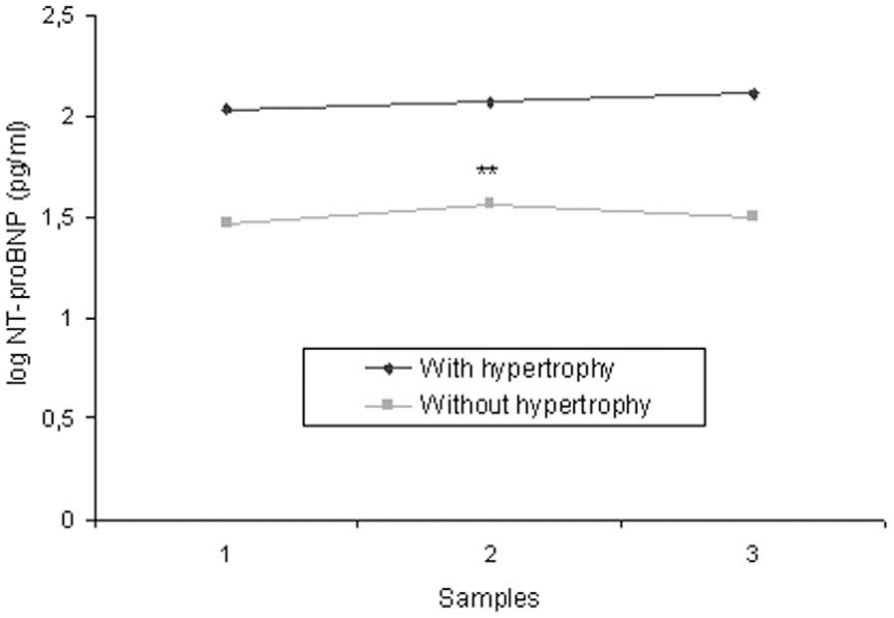
Serum logarithm of NT-proBNP levels during a 24-month follow-up. Measurements represent the median value at basal (1), stage I (2) and stage II (3). NT-proBNP, N-terminal pro-brain natriuretic peptide levels; stage I, 12-month follow-up; stage II, 24-month follow-up. **p<0.01 with respect to basal levels.

The baseline characteristics and natriuretic peptide serum levels of the hypertensive patients in the three stages according to hypertrophy are shown in Tables 1 (LVH group, n = 122) and 2 (non-LVH group, n = 98). Significant differences in blood pressure and total cholesterol levels were observed with respect to the basal stage in both groups. Body mass index, heart rate, biochemical values, left ventricular mass index and diastolic function variables did not show any statistical changes. In addition, significant differences were found in ejection fraction and NT-proBNP concentration in the non-LVH group. Figure 1 shows the mean of NT-proBNP serum levels over the entire study according to left ventricular hypertrophy. No differences were found with respect to the basal stage in the LVH group, but significant changes were observed in non-LVH subjects. NT-proBNP levels were increased in LVH hypertensive patients compared to patients without hypertrophy (p<0.0001).

**Table 1:** Clinical characteristics of patients with essential hypertension and left ventricular hypertrophy over the entire study (n = 122): basal, stage I = at 12 months, stage II = at 24 months.

Variable	Basal	Stage I	Stage II
Age (years)	65±13	66±13	67±12
SBP (mm Hg)	151±23	149±23	144±23‡
DBP (mm Hg)	86±11	85±12	82±11†
PP (mm Hg)	65±20	64±20	62±20†
Heart rate (bpm)	70±13	70±11	69±12
BMI (kg/m^2^)	31±5	31±5	32±5
GFR (ml/min/1.73 m^2^)	91±22	89±26	89±28
Total cholesterol (mg/dl)	212±36	204±34*	200±31†
Na (mEq/l)	140±5	141±3	141±3
Hemoglobin (mg/dl)	14±1	14±1	14±1
Diabetes mellitus (%)	18	21	22
EF (%)	58±5	58±5	59±5
E/A	0.84±0.25	0.84±0.21	0.82±0.19
Vp (cm/s)	44±10	46±12	46±11
DT (ms)	209±40	206±38	207±30
RVm	41±9	42±10	42±9
LVMI (g/m^2.7^)	63±15	64±14	64±15
Serum NT-proBNP (pg/ml)	102 (45–236)	92 (62–188)	114 (61–265)

Results are shown as mean (SD) or percentage of subjects. NT-proBNP levels are presented as the median and interquartile range.

BMI, body mass index; DBP, diastolic blood pressure; DT, deceleration time; E/A, flow velocity in early diastole and during atrial contraction ratio; EF, ejection fraction; GFR, glomerular filtration rate; LVMI, left ventricular mass index; NT-proBNP, N-terminal pro-brain natriuretic peptide levels; PP, pulse pressure; RVm, maximum longitudinal relaxation velocity of left ventricle; SBP, systolic blood pressure. Significant difference versus basal levels; Vp, mitral flow propagation velocity:

*p<0.05;

† p<0.01;

‡ p<0.0001.

**Table 2:** Clinical characteristics of patients with essential hypertension without left ventricular hypertrophy over the entire study (n = 98): basal, stage I = at 12 months, stage II = at 24 months.

Variable	Basal	Stage I	Stage II
Age (years)	53±11	54±10	55±12
SBP (mm Hg)	146±17	135±16‡	136±14‡
DBP (mm Hg)	88±11	83±10‡	83±9‡
PP (mm Hg)	58±14	52±12†	53±12†
Heart rate (bpm)	70±12	71±11	70±10
BMI (kg/m^2^)	28±3	28±3	28±3
GFR (ml/min/1.73 m^2^)	93±25	94±21	93±21
Total cholesterol (mg/dl)	214±40	206±33*	206±33*
Na (mEq/l)	141±2	140±5	139±7
Hemoglobin (mg/dl)	14±1	15±1	15±1
Diabetes mellitus (%)	17	20	21
EF (%)	59±5	59±5	61±5*
E/A	1.02±0.22	1.05±0.27	1.01±0.26
Vp (cm/s)	56±8	56±11	57±12
DT (ms)	186±28	183±22	184±22
RVm	50±9	53±11	54±12
LVMI (g/m^2.7^)	39±6	39±6	39±9
Serum NT-proBNP (pg/ml)	33 (16–54)	39 (27–60)†	37 (18–61)

Results are shown as mean ± SD or percentage of subjects. NT-proBNP levels are presented as the median and interquartile range.

BMI, body mass index; DBP, diastolic blood pressure; DT, deceleration time; E/A, flow velocity in early diastole and during atrial contraction ratio; EF, ejection fraction; GFR, glomerular filtration rate; LVMI, left ventricular mass index; NT-proBNP, N-terminal pro-brain natriuretic peptide levels; PP, pulse pressure; RVm, maximum longitudinal relaxation velocity of left ventricle; SBP, systolic blood pressure. Significant difference versus basal levels; Vp, mitral flow propagation velocity:

*p<0.05;

† p<0.01;

‡ p<0.0001.

**Figure 2:**
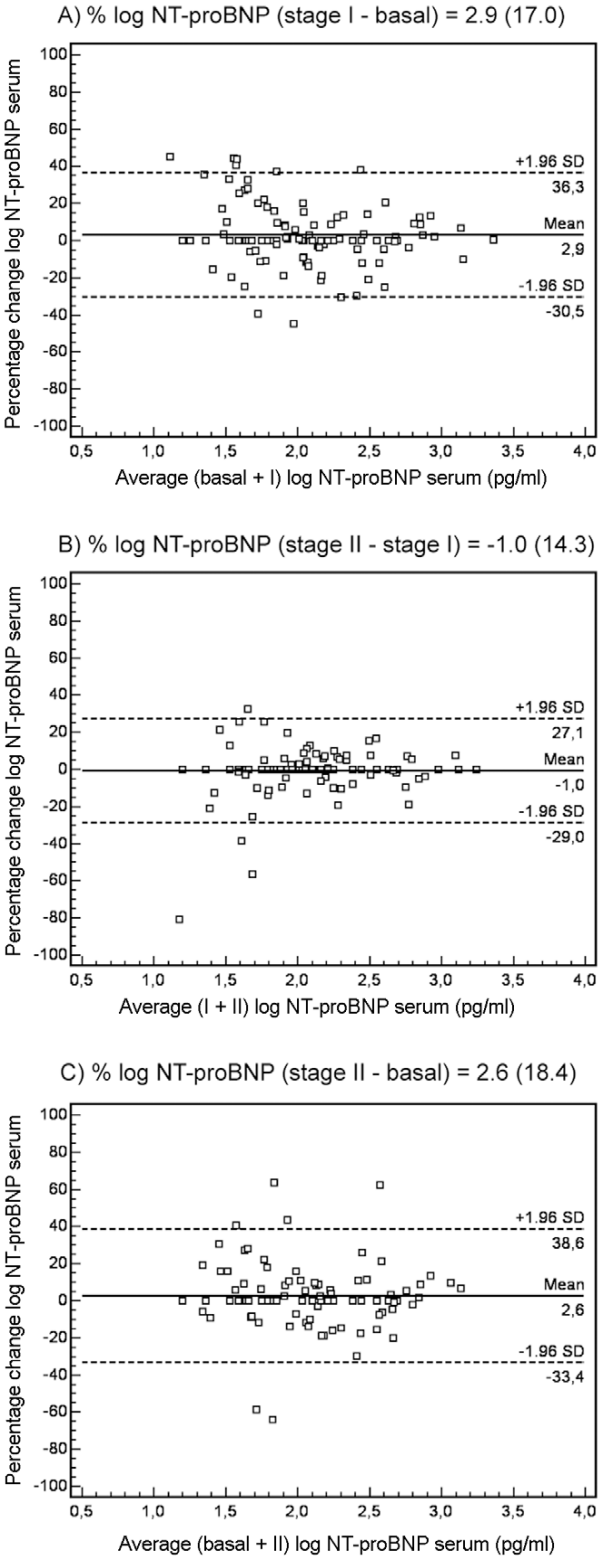
Bland-Altman plots for changes in NT-proBNP serum levels in hypertensive patients with hypertrophy. Bland-Altman plot showing agreement between the logarithm of NT-proBNP levels percentage change against the average of the logarithm of NT-proBNP levels in basal + stage I (A), stage I + stage II (B) and basal + stage II (C). The solid line represents the mean of the percentage change. The dashed lines define the limits of agreement (standard deviation of percentage of change ×1.96 SD). NT-proBNP, N-terminal pro-brain natriuretic peptide levels; SD, standard deviation; stage I, 12-month follow-up; stage II, 24-month follow-up.

**Figure 3:**
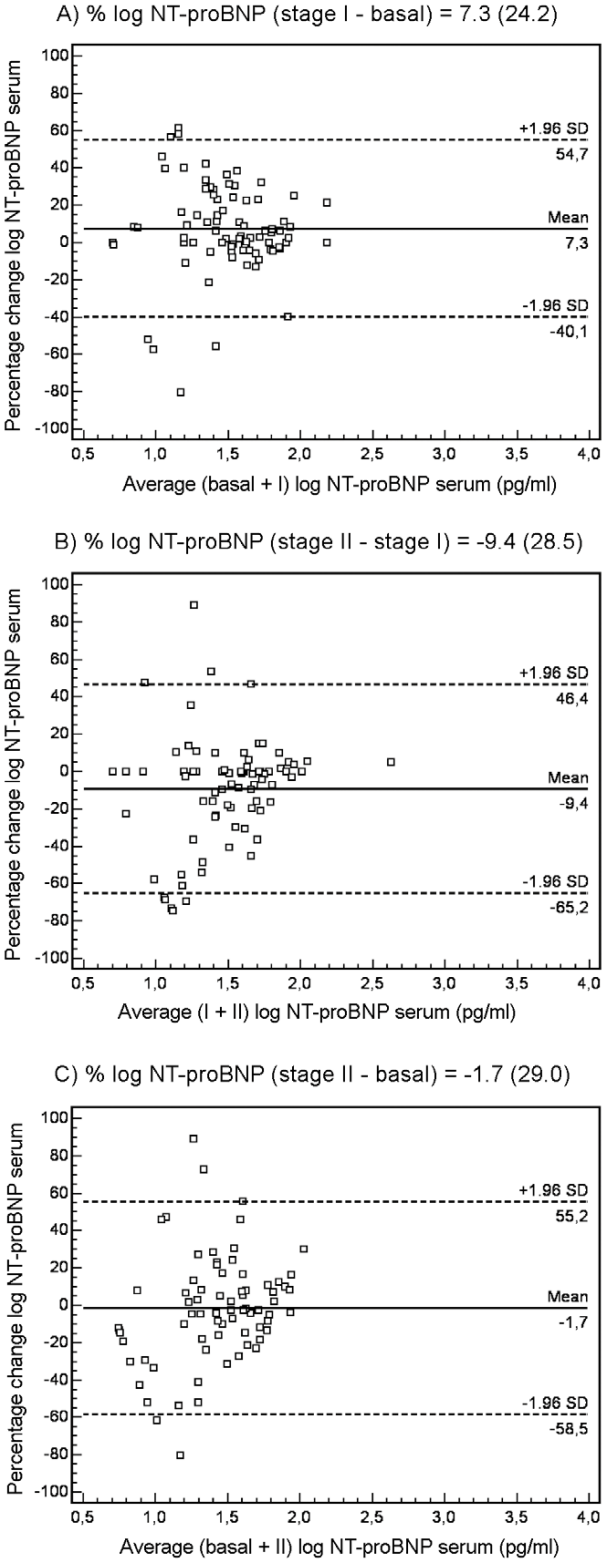
Bland-Altman plots for changes in NT-proBNP serum levels in hypertensive patients without hypertrophy. Bland-Altman plot showing agreement between the logarithm of NT-proBNP levels percentage change against the average of the logarithm of NT-proBNP levels in basal + stage I (A), stage I + stage II (B) and basal + stage II (C). The solid line represents the mean of the percentage change. The dashed lines define the limits of agreement (standard deviation of percentage of change ×1.96 SD). NT-proBNP, N-terminal pro-brain natriuretic peptide levels; SD, standard deviation; stage I, 12-month follow-up; stage II, 24-month follow-up.

The reproducibility of NT-proBNP measurements was better in patients with LVH than in those without LVH. Figure 2 shows the Bland-Altman plots for changes in NT-proBNP serum levels in patients with LVH over each of the intervals studied (A: stage I – basal; B: stage II – stage I; C: stage II – basal). In the A interval, 93.4% of patients fell within 1.96 SD of the mean. The mean ± SD percentage of change of peptide levels agreement was 2.9±17.0, with a CR of 33%, **and the mean ± SD absolute change was 106±107 pg/ml.** In the B interval, 96.7% of patients fell within the range of 1.96 SD, with a mean percentage of change of -1±14.3, CR of 28%, **and the mean value of absolute change was 87±88 pg/ml.** Finally, in the C interval, 95.1% of patients fell within 1.96 SD of the mean, with a mean change of 2.6±18.4, CR of 36% **and the mean value of absolute change was 110±111 pg/ml**. Figure 3 shows the changes in NT-proBNP serum levels in patients without LVH in the A, B and C intervals. The percentages of patients within 1.96 SD of the mean were 92.9%, 90.1% and 94.9%, respectively. The values of the mean ± SD percentage change and CR were 7.3±24.2 with a CR of 47%; −9.4±28.5 with a CR of 56%; and −1.7±29.0 with a CR of 57%, respectively. **The mean value of absolute change was 32±38 pg/ml, 29±43 pg/ml and 31±45 pg/ml, respectively.** **For the group of hypertensive patients with hypertrophy, percentage CVt and CVi in the A interval was 12.3% and 12.1%, in the B interval 12.5% and 12.3%, and in the C interval 14.7% and 14.5%, respectively; and the corresponding RCVs were 34%, 35% and 41%. For the group of hypertensive patients without hypertrophy, percentage CVt and CVi in the A interval was 21.1% and 20.9%, in the B interval 28.5% and 28.4%, and in the C interval 29.1% and 28.9%, respectively; and the corresponding RCVs were 58%, 79% and 81%.** In addition, when we analyzed the correlation between the different NT-proBNP measurements, a more elevated coefficient of correlation was obtained in the LVH group than in patients without LVH: basal versus stage I (r = 0.79, p<0.0001 and r = 0.59, p<0.0001) and stage I versus stage II (r = 0.86, p<0.0001 and r = 0.56, p<0.0001).

Finally, when we calculated the correlation between NT-proBNP levels and the concentrations of the inflammatory markers analyzed, a good coefficient of correlation was obtained in the group of hypertensive patients with LVH. Serum levels of NT-proBNP significantly correlated with plasma concentrations of sTNF-R1 (p<0.0001) and IL-6 (p<0.01) during follow-up. However, in the group of hypertensive patients without LVH this relationship was lower (Table 3). In addition, a multivariate linear regression analysis was used to test the independent predictive power of these inflammatory mediators (adjusted for age, gender, **blood pressure, total cholesterol** and treatment) on log-transformed NT-proBNP in the hypertensive patients with LVH. In each of the intervals studied the best model included log transformed sTNF-R1 as independent factor (Table 4).

**Table 3:** Pearson's coefficient (*r*) between log-transformed serum NT-proBNP and inflammatory markers according to left ventricular hypertrophy over the entire study: basal, stage I = at 12 months, stage II = at 24 months.

	With LVH (n = 122)			Without LVH (n = 98)		
	log NT-proBNP (pg/ml)			log NT-proBNP (pg/ml)		
	Basal	Stage I	Stage II	Basal	Stage I	Stage II
log sTNF-R1 (pg/ml)	0.44‡	0.52‡	0.45‡	0.22*	0.24*	0.23*
log IL-6 (pg/ml)	0.29†	0.31†	0.28†	0.11	0.18	0.14

log NT-proBNP, log-transformed N-terminal pro-brain natriuretic; LVH, left ventricular hypertrophy; log IL-6, log-transformed interleukin-6; log sTNF-R1, log-transformed soluble tumor necrosis factor receptor 1.

*p<0.05;

† p<0.01;

‡ p<0.0001.

**Table 4:** Multivariate linear regression to evaluate the association of log-transformed NT-proBNP with inflammatory markers in hypertensive patients with hypertrophy.

	Basal			Stage I			Stage II		
	B	S.E.	p Value	B	S.E.	p Value	B	S.E.	p Value
Age	0.015	0.003	0.0001	0.014	0.003	0.0001	0.026	0.004	0.0001
Gender	−0.074	0.070	0.293	−0.057	0.073	0.435	−0.009	0.092	0.924
SBP	0.003	0.002	0.175	0.000	0.002	0.994	−0.002	0.004	0.620
DBP	0.000	0.004	0.965	0.004	0.004	0.334	−0.004	0.006	0.561
Total cholesterol	0.000	0.001	0.955	0.000	0.001	0.945	−0.001	0.001	0.380
ARA II	−0.023	0.081	0.775	−0.033	0.087	0.704	−0.060	0.120	0.621
Diuretics	−0.057	0.067	0.392	−0.026	0.067	0.696	−0.008	0.090	0.928
ACE inhibitors	0.005	0.085	0.950	−0.002	0.090	0.981	−0.024	0.121	0.844
β-blockers	0.302	0.075	0.0001	0.238	0.078	0.003	0.275	0.080	0.003
CCBs	0.072	0.080	0.370	−0.092	0.091	0.317	0.130	0.118	0.275
log sTNF-R1	0.759	0.187	0.0001	0.745	0.177	0.0001	0.537	0.177	0.025
log IL-6	0.070	0.108	0.517	−0.211	0.242	0.386	−0.051	0.182	0.779

Basal: r^2^ of 0.54 (p<0.0001); Stage I: r^2^ of 0.64 (p<0.0001); Stage II: r^2^ of 0.57 (p<0.0001). ACE, angiotensin converting enzyme; ARA II, antagonists receptors angiotensin type II; CCBs, calcium-channel blockers; DBP, diastolic blood pressure; log IL-6, log-transformed interleukin-6; log sTNF-R1, log-transformed soluble tumor necrosis factor receptor 1; SBP, systolic blood pressure.

## Discussion

In a homogeneous and representative group of the hypertensive population we found good stability of NT-proBNP levels in patients with clinically and functionally stable hypertension and LVH. This is the first study to monitor changes in serum NT-proBNP concentration over time in asymptomatic clinically stable patients with essential hypertension, and this would allow us to know its usefulness in the clinical setting. In addition, we found a significant relationship between this natriuretic peptide and the inflammatory status, especially in the group of hypertensive patients with LVH.

The presence of left ventricular hypertrophy adversely affects the prognosis of patients with arterial hypertension. NT-proBNP can predict outcome in patients with hypertension and LVH without left ventricular dysfunction or renal disease, independently of traditional cardiovascular risk factors including high blood pressure, **renal function and electrocardiographic indexes** [6]. **The knowledge of variations in NT-proBNP levels is necessary before this peptide can be used clinically as a tool to monitor patients. Several works have evaluated the biological variation of BNP and NT-proBNP concentrations in both patients with chronic heart failure and healthy people over a short (within a day, week to week and month to month), intermediate (1-month, 2-month, and 3-month), and long-term (year-to-year) interval of time** [**8]**–[**13**] **. To date, there are no data on the changes in serum NT-proBNP levels over time in patients with essential hypertension.** In this work, we evaluated the biological variation of serum NT-proBNP levels in a 24-month follow-up of clinically stable asymptomatic hypertensive patients.

In our 220 patients with clinically and functionally stable hypertension there were neither cardiovascular events nor differences in ventricular function, but ejection fraction showed significant variation in stage II with respect to basal values in the non-hypertrophic group (61% versus 59% mean) and we think that this minimal change could be attributed to the methodology used [28]. Moreover, we found significant differences for values of blood pressure and total cholesterol in both groups, these variables decreasing over time probably as a consequence of the treatment. At first, we could think that these changes in blood pressure values affect the natriuretic peptide concentrations during follow-up. However, **the results of the present study** and previous works have shown that blood pressure (systolic and diastolic) and total cholesterol levels were not independent predictors of natriuretic peptide levels in hypertensive patients [29], [30]. **In addition, Frankenstein** ***et al.*, showed that the individual biological variability of NT-proBNP does not appear to be influenced by known confounders, such as sex, age, weight or waist circumference, ejection fraction, or renal function** [**11**] **.**

When we compared the levels of NT-proBNP over time according to LVH, we only found significant changes in stage II with respect to basal values in the non-LVH group, but in LVH patients NT-proBNP levels remained stable. Furthermore, good correlation was obtained between NT-proBNP concentrations at the three stages over the entire study, the correlation coefficients being higher for the group of hypertensive patients with hypertrophy. These findings also prove higher stability of NT-proBNP levels in LVH patients with respect to non-LVH.

Another consideration of natriuretic peptide stability is that the percentage change in our patients with LVH over the entire study (24-month follow-up) presented an SD below 19%. In the non-LVH group the SD was lower than 29%. **In addition, biological variability and the resulting RCVs were lower in hypertrophic subjects.** This difference between results may occur because the peptide levels are closely regulated by specific pathophysiological mechanisms, and although stretch of cardiac myocytes is considered the main stimulus for NT-proBNP secretion, certain physiology conditions can also stimulate peptide release [31], [32]. In other words, in patients presenting LVH there is a predominant stimulus for the synthesis of NT-proBNP which is cardiac wall stress [33], however, in patients without ventricular hypertrophy the peptide concentrations found in the bloodstream could be a consequence of diverse and variable physiological conditions [34]. Furthermore, NT-proBNP immunoassay methods had some variability and this may induce higher % variations in patients showing lower peptide values.

One important clinical consequence of our study is the establishment of an NT-proBNP percentage change, from which we can monitor the progress of these patients. Thus, we suggest that all NT-proBNP measured variations, with a coefficient of reproducibility (>1.96 SD percentage change) above 33% in a 12-month follow-up and 36% in a 24-month follow-up, could be considered an increase in potential risk. In a previous study, our group established a similar coefficient of reproducibility in patients with heart failure (25%) in a 24-month follow-up [13]. Nowadays, NT-proBNP usefulness to monitor heart failure patients is well established [35]. **In our group of hypertrophic patients RCVs were 35% in a 12-month follow-up and 41% in a 24-month follow-up, meaning that concentrations had to increase or decrease by these percentages to be assessed as different from the previous measurement, with a 5% error probability for falsely assessing a change as significant, being considered of possible prognostic importance** . **These results are in concordance with an analysis performed on stable heart failure patients** [**11**] **. In contrast, results of prior studies found RCVs around 100%** [**9]**, [**10**] **. The clinical relevance of these large RCVs are controversial, but the key to understanding these results is the definition of stability of heart failure and also the estimation of CVs.** We think there could be a clinical shift in using natriuretic peptides to change management of those with hypertension and LVH over time. **The evidence has clearly demonstrated that NT-proBNP is a powerful predictor of mortality and cardiovascular risk in hypertensive patients.** **This suggests the importance of NT-proBNP in the assessment of these patients to perform a more accurate risk evaluation and in the future to possibly lead to an NT-proBNP guided therapy able to get a more favorable clinical outcome.**

Up-regulation and production of cytokines represent an intrinsic or an innate stress response against myocardial injury and its elevation predicts short- and long-term incidence of cardiovascular adverse events [14], [15]. In a previous report, our group showed that the levels of different cytokines were increased in hypertensive patients with LVH. Plasma cytokine levels, such as IL-6 and sTNF-R1, were correlated with left ventricular mass index, but when a logistic regression was performed to predict hypertrophy, only sTNF-R1 was independent predictor [16]. Some authors have shown that NT-proBNP serum levels are increased in hypertensive patients with LVH [6], [29]. In this sense, the results of the present study are in concordance with these findings. We found a good relationship between immune system activation and natriuretic peptide levels in hypertensive patients with LVH, specifically, a strong correlation between NT-proBNP and sTNF-R1 concentrations. However, the molecular mechanisms by which natriuretic peptides and inflammatory mediators are related are uncertain. Ventricular wall stress is the principal factor stimulating brain natriuretic peptide synthesis and release from cardiomyocytes [36], [37]. Furthermore, this stress also produces an increase in cytokine levels, and the cytokines amplify the signal, because they have a pleiotropic effect [38]. An interesting finding is that the elevation in cytokine expression precedes the increase in natriuretic peptides and collagen expression in a rodent model of myocardial infarction [39]. These results could be translated to other pathological conditions involving heart diseases. As inflammatory mediators are elevated in hypertensive patients with LVH, this might explain in part the significant elevation of NT-proBNP levels compared with patients without hypertrophy and therefore, this also implies new therapeutic options to improve hypertension treatment in the future. However, studies on the relationship between inflammatory markers and natriuretic peptides are limited. In fact, to the best of our knowledge, cytokine levels have never been correlated with NT-proBNP concentrations in hypertensive patients. Thus, more work is necessary to fully understand the role of the cytokines studied in the activation of the neurohormonal system.

One limitation of this study is that our patients were on medication and it is known that NT-proBNP values could be affected by treatment with diuretics, angiotensin-converting enzyme inhibitors, angiotensin II receptor blockers, or beta-blockers [40]–[42]. Nevertheless, this circumstance makes it easier to extrapolate our data to the clinical practice. Moreover, we have to admit that a larger group of patients would have provided additional information. However, the strict inclusion-exclusion criteria give our results greater value.

An important consideration is that we selected patients with clinically stable hypertension without clinical or functional changes, but we cannot rule out the possibility of subtle changes in neurohormonal and immunology systems that might potentially influence the variability of natriuretic peptide levels. However, we think that because of this, our data are more useful for judging the clinical variations in NT-proBNP levels, and they have evident practical application.

Although we think that natriuretic peptides can be useful to monitor hypertensive patients, this study has not been designed to establish an optimal frequency of checking lab values. In addition, it would be interesting from the clinical point of view to know whether the variability in NT-proBNP levels is associated to events occurring after the 2 years of the study in both the populations studied (hypertensive patients with and without hypertrophy), however, this study was designed and funded only for a 2-year follow-up. Further studies would most definitely help clarify these points.

Another potential limitation is that although echocardiography-standardized techniques have been shown to be a more sensitive tool for detecting LVH than electrocardiographic measurements [43], the variability of this technique is higher than the variability using magnetic resonance imaging. However, in this study a specialized, blinded, single cardiologist performed the echocardiographic analyses to measure the left ventricular mass to minimize variability.

In conclusion, this work shows that there is good stability in NT-proBNP levels in a 24-month follow-up study of asymptomatic patients with clinically and functionally stable hypertension and LVH. As a consequence, **and taking into account its strong predictive power of mortality and cardiovascular risk in hypertension**, assessment of NT-proBNP concentrations may be a useful tool for monitoring the follow-up of hypertensive patients with hypertrophy. Measured variations in peptide levels, exceeding **35%** in a 12-month follow-up and **41%** in a 24-month follow-up, may indicate an increase in cardiovascular risk, and therefore implies adjustment in the medical treatment. In addition, this study shows a link between neurohormonal and inflammatory activation in these patients.
